# Depletion of Human Papilloma Virus E6- and E7-Oncoprotein-Specific T-Cell Responses in Women Living With HIV

**DOI:** 10.3389/fimmu.2021.742861

**Published:** 2021-10-25

**Authors:** Wilbert Mbuya, Kathrin Held, Ruby D. Mcharo, Antelmo Haule, Jacklina Mhizde, Jonathan Mnkai, Anifrid Mahenge, Maria Mwakatima, Margareth Sembo, Wolfram Mwalongo, Peter Agrea, Michael Hoelscher, Leonard Maboko, Elmar Saathoff, Otto Geisenberger, France Rwegoshora, Liset Torres, Richard A. Koup, Arne Kroidl, Mkunde Chachage, Christof Geldmacher

**Affiliations:** ^1^ National Institute for Medical Research – Mbeya Medical Research Centre (NIMR-MMRC), Mbeya, Tanzania; ^2^ Division of Infectious Diseases and Tropical Medicine, University Hospital, Ludwig Maximilian University (LMU) Munich, Munich, Germany; ^3^ German Center for Infection Research (DZIF), Partner Site Munich, Munich, Germany; ^4^ Tanzania Commission for AIDS (TACAIDS), Dar es Salaam, Tanzania; ^5^ Pathology Department, Mbeya Zonal Referral Hospital, Mbeya, Tanzania; ^6^ Vaccine Research Centre, National Institute for Allergy and Infectious Diseases, National Institutes of Health, Bethesda, MD, United States; ^7^ Microbiology and Immunology Department, University of Dar es Salaam -Mbeya College of Health and Allied Sciences (UDSM-MCHAS), Mbeya, Tanzania

**Keywords:** HPV, HIV, T-cell response, oncoprotein, cervical cancer

## Abstract

**Background:**

Cervical cancer - caused by persistent High Risk Human Papilloma Virus (HR HPV) infections - is the second most common cancer affecting women globally. HIV infection increases the risk for HPV persistence, associated disease progression and malignant cell transformation. We therefore hypothesized that this risk increase is directly linked to HIV infection associated dysfunction or depletion of HPV-oncoprotein-specific T-cell responses.

**Methods:**

The 2H study specifically included HIV+ and HIV- women with and without cervical lesions and cancer to analyze HPV oncogene-specific T cell responses in relation to HPV infection, cervical lesion status and HIV status. Oncoprotein E6 and E7 specific T-cell responses were quantified for the most relevant types HPV16, 18 and 45 and control antigens (CMV-pp65) and *M.tb*-PPD in 373 women, using fresh peripheral blood mononuclear cells in an IFN-γ release ELISpot assay.

**Results:**

Overall, systemic E6- and E7-oncoprotein-specific T-cell responses were infrequent and of low magnitude, when compared to CMV-pp65 and *M.tb*-PPD (p < 0.001 for all HR HPV types). Within HIV negative women infected with either HPV16, 18 or 45, HPV16 infected women had lowest frequency of autologous-type-E6/E7-specific T-cell responses (33%, 16/49), as compared to HPV18 (46% (6/13), p = 0.516) and HPV45 (69% (9/13), p = 0.026) infected women. Prevalent HPV18 and 45, but not HPV16 infections were linked to detectable oncoprotein-specific T-cell responses, and for these infections, HIV infection significantly diminished T-cell responses targeting the autologous infecting genotype. Within women living with HIV, low CD4 T-cell counts, detectable HIV viremia as well as cancerous and precancerous lesions were significantly associated with depletion of HPV oncoprotein-specific T-cell responses.

**Discussion:**

Depletion of HPV-oncoprotein-specific T-cell responses likely contributes to the increased risk for HR HPV persistence and associated cancerogenesis in women living with HIV. The low inherent immunogenicity of HPV16 oncoproteins may contribute to the exceptional potential for cancerogenesis associated with HPV16 infections.

## Introduction

Cervical cancer, typically caused by persistent high risk human papilloma virus (HR HPV) infections, is the second most frequent cancer affecting women worldwide, with 570,000 new cases and 311,000 deaths per year (1). Eighty percent of worldwide cervical cancer cases occur in low-income countries, with sub-Saharan African (SSA) countries being amongst the most heavily affected (2). HIV infection dramatically increases the risk for HPV infection, persistence and rapid progression to cervical cancer (3–7), resulting in elevated cervical cancer incidences in regions with high HIV prevalence rates.

HR HPV infections are common and typically cause transient infections that are cleared within two years (8). However, HR HPVs can also establish persistent infections that cause cervical dysplasia which can subsequently progress to squamous cell carcinoma (SCC) (9, 10). HPV16, as well as the types HPV18 and 45, globally and within East Africa cause the vast majority of SCC (11; Mcharo et al., 2021, in press).

T cells are thought to play a key role in clearing HPV infection and disease. T-cell infiltration into HPV-associated skin and genital warts has been linked to regression of the warts (12, 13). Similarly, infiltrating T cells are predominant in cervical intraepithelial lesions and cancers (14–16). HPV16 E6- or E7-specific T-cell responses have been detected more frequently in women clearing HPV16 infections than in those with persistent infection (17–19). Furthermore, a number of studies report a decrease in the frequency of systemic HPV-E6/E7-specific CD4 and CD8 T-cell responses as cervical lesions progress to cancer (20–22). However, overall, systemic HPV-specific T-cell responses are infrequent and difficult to measure (23) and hence difficult to study.

While the precise mechanisms of HIV pathogenesis remain subject of intense investigations, the depletion of pathogen-specific T-cell responses has been linked to the risk for disease by opportunistic infections such as *Mycobacterium tuberculosis* (*M.tb*), cytomegalovirus (CMV) and HR HPVs in people living with HIV (22, 24, 25). For instance, *M.tb*-specific Th1 cells are preferentially depleted early during the course of HIV infection, and tuberculosis is often the first opportunistic infection affecting HIV+ patients (26–28). Likewise, eventual loss of CMV- and Epstein-Bar Virus (EBV)-specific CD4 T cells precede CMV end organ disease and development of AIDS related EBV-associated lymphomas, respectively (29, 30). Antiretroviral therapy (ART) interferes with HIV replication, reverses CD4 T-cell depletion and improves memory T-cell responses against most common opportunistic pathogens (31–33). This ART-induced immune reconstitution protects against most AIDS-defining diseases and as a consequence, prolongs the life of HIV+ individuals (34). The incidence of malignancies caused by HR HPV, however, has not decreased during the era of ART - a phenomenon that is still not fully understood (35). Amongst other factors, the level of immune reconstitution is influenced by differences in ART adherence between individual patients and the stage of HIV disease progression at which ART had been initiated. Guidelines on the use of antiretroviral drugs for treating and preventing HIV infection by WHO define immunological failure as CD4 T-cell counts of 250 cells/mm^3^ or less proceeded by clinical failure, and virological failure is defined as two sequential viral loads (VL) levels of 1000 or more copies/mL within 3 months (36).

In this study, we addressed the hypothesis that HIV infection depletes HR HPV-specific T-cell responses as a possible underlying mechanism for increased HPV persistence and accelerated cancerogenesis in women living with HIV. We therefore examined the frequency and magnitude of HR HPV-specific T-cell responses determined by IFN-γ release ELISpot assay in relation to HR HPV and HIV infection, ART status, systemic HIV VL levels and CD4 T-cell count.

## Material and Methods

### Study Population

The women studied herein were part of the prospective, longitudinal case-control 2H study. This study was designed to dissect the influence of HIV on HPV infection and carcinogenesis and was conducted from 2013 to 2020 in Mbeya, South-West Tanzania. HIV+ and HIV- women above the age of 18 attending the Cervical Carcinoma Screening (CCS) at the Mbeya Zonal Referral Hospital HIV Care and Treatment Centre, at the META Gynecological Outpatient Department of the Mbeya Zonal Referral Hospital, the Matema Lutheran Hospital as well as health care facilities in the greater Mbeya urban and regional area with CCS services were recruited and screened for cervical lesions, cancer and for HIV infection. Selected volunteers were then fully enrolled within 2 month after the screening visit to allow for immunological analyses and collection of peripheral blood mononuclear cells (PBMC) for specific study groups defined by HIV infection and the presence or absence of high and low grade intraepithelial lesions and cancer of the cervix. The primary focus of the immunological analyses was to determine frequency and magnitude of systemic HPV-oncoprotein-specific T-cells responses in relation to HIV infection and cervical lesion status. Therefore, IFN-γ release ELISpot assay results from 373 well characterized 2H study participants were analysed cross-sectionally. A detailed description of the clinical parameters of these study volunteers is shown in **Table 1**. The quantification of HPV-specific T-cell responses by IFN-γ release ELISpot is described in detail below.

**Table 1:** Clinical data of study participants stratified by HIV status.

	Total	HIV negative	HIV positive	p value
Median age (interquartile range)	41 (34 – 51)	46 (37 – 60)	39 (32 – 45)	<0.001^a^
cytohistological diagnosis (n) †	n = 373	n = 171 (47%)	n = 202 (53%)	
SCC	126	75 (60%)	51 (40%)	<0.001^b^
HSIL/CIN2+	44	10 (23%)	34 (77%)	0.001 ^b^
LSIL/CIN1	39	7 (18%)	32 (82%)	<0.001 ^b^
no lesion	162	78 (48%)	84 (52%)	0.464 ^b^
AGC	2	1 (50%)	1 (50%)	
Molecular HPV diagnosis (n)	n = 343	n = 149	n = 194	
HPV 16+	107	49 (46%)	58 (54%)	0.559 ^b^
HPV 18+	49	13 (27%)	36 (73%)	0.012 ^b^
HPV 45+	38	16 (42%)	22 (58%)	1.000 ^b^
Any HR-HPV+	243	88 (36%)	155 (64%)	<0.001 ^b^
HR-HPV-	100	61 (61%)	39 (39%)	<0.001 ^b^
HPV-	89	56 (63%)	33 (37%)	<0.001 ^b^
ART status, CD4 and HIV VL counts (n)	n = 373	n = 171	n = 202	
On ART	n/a.	n/a.	75% (139/185)	
HIV+ with ≤ 250 CD4 T cells/mm^3^	n/a.	n/a.	34% (61/177)	
HIV+ with ≥ 1001 HIV RNA copies/ml	n/a.	n/a.	34% (61/179)	

^†^pathology diagnosis based on cytology and confirmed by histology. SCC, cervical cancer; HSIL, high grade intraepithelial lesion; CIN, cervical intraepithelial neoplasia; LSIL, low grade intraepithelial lesion; ART, antiretroviral therapy; AGC, Atypical glandular cells; n/a, not applicable. For statistical analysis: ^a^students t-test; ^b^Fisher’s exact test.

### Ethical Consideration

Ethical clearance was obtained from the Mbeya Medical Research and Ethics review Committee (MRH/R.10/8/Vol. VI/107), the Tanzanian National Health Research Ethics Committee (NIMR/HQ/R.8a/Vol. IX/1422) and the Ethics Committee of the Medical Faculty of University of Munich (project ID: 308-11) before commencement of the 2H study. All study participants were fully briefed on study procedures, and signed informed consent was obtained from all study participants before enrolment. All procedures pertaining to clinical examination of the volunteers and sample collection for laboratory assays were performed by certified clinicians and in adherence to the Tanzanian National Guidelines.

### Specimen Collection for Clinical and Immunological Assessments

Peripheral blood for absolute CD4 T-cell counts and HIV VL quantification was collected into EDTA tubes (BD) and peripheral blood mononuclear cells (PBMCs) for the quantification HPV-specific T-cell response by the IFN-γ release ELISpot assay were isolated by Ficoll density gradient centrifugation of whole blood collected into ACD tubes (BD) using the manufacturer’s protocol. Cervical cells for HPV genotyping were obtained from the endocervix by gently rotating a cytobrush (Solann) 360 degrees around the endocervical wall. The brush was immediately transferred into a falcon tube with 5 mL PreservCyt cell collection media (Roche). Pap smear for Papanicolaou testing was collected by gently rotating an Ayres spatula in the ectocervix. In cancer suspicious cases, a biopsy was taken for further histological diagnosis at the pathology department of the Mbeya Zonal Referral Hospital.

### HIV Diagnosis, ART Status Determination, CD4 T-Cell Counts, and HIV VL Quantification

HIV testing was done at recruitment into the 2H study using two independent HIV-Rapid tests: First by Determine HIV1/2 (Abbott Laboratories, South Africa) and then confirmed with Uni-Gold HIV Rapid Test (Trinity Biotech, South Africa). For HIV+ women, absolute CD4 T-cell counts were analysed from peripheral blood samples using BD Trucount tubes (BD) and acquired on a BD FACSCalibur (BD), while HIV VL was quantified using COBAS^®^ AmpliPrep/COBAS^®^ TaqMan^®^ HIV-1 Test (Roche), both according to manufacturer’s instructions. Information on ART status was obtained by interviewing the volunteer.

### HPV Genotyping

Cervical cells were collected and stored as described above in PreservCyt^®^ Solution (Roche) and subjected to HPV genotyping using Roche linear array genotyping kit following the manufacturer’s instructions. This assay detects thirty-seven HPV (including all HR HPV) genotypes: HPV types 6, 11, 16, 18, 26, 31, 33, 35, 39, 40, 42, 45, 51, 52, 53, 54, 55, 56, 58, 59, 61, 62, 64, 66, 67, 68, 69, 70, 71, 72, 73, 81, 82, 83, 84, IS39 and CP6108. The following analyses focused mainly on HPV types 16, 18 and 45.

### Diagnosis of Cervical Pathology

Screening for cervical cancer and lesions was performed by visual inspection during the clinical assessment and by routine cytology using Papanicolaou testing. In cases where more detailed histologic diagnosis was required, biopsies were collected, and Hematoxylin & Eosin staining was performed at the pathology department of the Mbeya Zonal Referral Hospital (MZRH) Pathology department according to national guidelines. Cervical pathology results were reported as per the Bethesda system for reporting cervical or vaginal diagnoses (37).

### Antigens for *In Vitro* Restimulation of PBMCs

The following synthetic peptides were used for *ex vivo* re-stimulation of PBMCs: 15mer peptide pools overlapping by 11 amino acids for oncoproteins E6 and E7 specific to HPV16, 18 and 45 (Peptides and Elephants) and purified protein derivative from *M.tb* (*M.tb*-PPD: Staten Serum Institute) as well as CMV phosphoprotein 65 (CMV-pp65) overlapping peptide pools (Peptides and Elephants) were used to assess *M.tb*- and CMV-specific systemic T-cell responses. Phytohemagglutinin (PHA) (Sigma) was used as a positive control while complete media only [RPMI-1640 with 0.5% penicillin-streptomycin, 1% HEPES and 10% inactivated fetal bovine serum (FBS)] was used as negative control to determine assay background signal.

### Quantification of HPV-Oncoprotein-Specific T-Cell Responses by IFN-γ Release ELISpot

**Figure 1:**
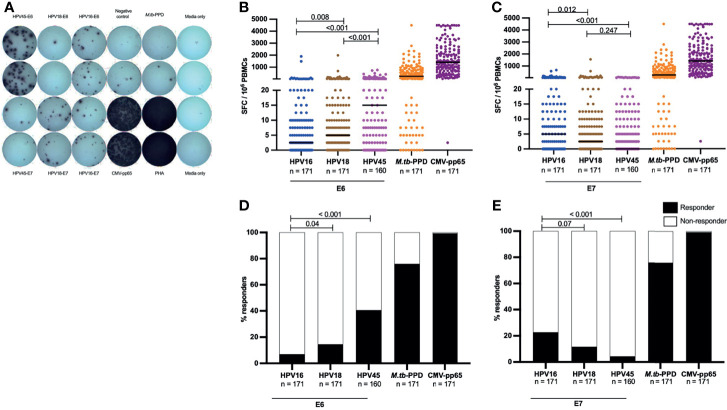
HR HPV oncoproteins have a low inherent systemic immunogenicity in HIV- women. A representative image of an IFN-γ release ELISpot assay plate **(A)**, showing T-cell reactivity against E6 and E7 oncoproteins for HPV16, 18 and 45, and responses against CMV-pp65, Mtb-PPD, and control wells (negative control wells (complete media + PBMCs), media only wells and positive (PHA) control wells) is shown in **(A)**. The magnitude of T-cell reactivity against both HPV oncogenes, as well as *M.tb*-PPD and CMV-pp65 is given in SFC/10^6^ PBMCs. PBMCs were stimulated with E6 **(B)** and E7 **(C)** HPV16, 18, 45 type specific oncoproteins, and control peptides *M.tb*-PPD and CMV-pp65 overnight and SFC/10^6^ PBMCs were recorded for each sample. Each dot represents one study volunteer and total numbers of women analysed is stated in the x-axis legend. Median SFC/10^6^ PBMCs is indicated by a black line. Statistical analysis was performed using the Mann-Whitney U-test. The proportion of responders for E6 **(D)** and E7 **(E)** as well as *M.tb*-PPD and CMV-pp65 are shown as percentage. The black bars indicate the proportion of individuals with a response while the white bar represent the proportion of individuals without a response, totaling to 100%. The n is given in the figure legend. Statistical analysis was performed using Fisher’s exact test, respective p-values are shown in the graph.

Pathogen-specific T cells were quantified by IFN-γ release ELISpot assay. 96 well plates (MultiScreen_HTS_ IP filter, 0.45 µm, Millipore) were pre-wetted 4 times with 200 μl sterile phosphate buffer saline (PBS) and coated with 50μl of anti-human IFN-γ capture antibody (5 μg/ml, clone 1-D1K, Mabtech). To allow the antibody to bind, the plates were placed at 4°C overnight. Before seeding cells, the plates were washed 4 times with sterile PBS to remove any unbound capture antibody and then blocked for 30 minutes with 200 μl complete medium [RPMI-1640 with 0.5% penicillin-streptomycin, 1% HEPES and 10% inactivated fetal bovine serum (FBS)]. 200,000 freshly isolated PBMCs were plated per well in duplicates and pathogen-specific antigens added as follows: 2μg/ml for each overlapping peptide of E6 and E7 for HPV16, 18 and 45; 2μg/ml for each peptide of CMV-pp65; 10μg/ml of *M.tb*-PPD and 40μg/ml of phytohemagglutinin (PHA). Complete media added to cells served as negative control (background). The plate was then incubated at 37°C and 4.5% CO_2_ for 20 hours. After incubation, the plate was washed 5 times with PBS, and finally 100 μl of biotinylated anti-IFN-γ monoclonal antibody (1μg/ml, clone 7-B6-1, Mabtech) in PBS containing 0.5% FBS was added. The plate was then incubated in the dark at room temperature for 2 hours. This was followed by 5 times washing with PBS and addition of 100μl of streptavidin - alkaline phosphatase conjugate (Mabtech) at a concentration of 1μg/ml in PBS with 0.5% FBS and then a further incubation for 1 hour. The plates were then washed 5 times to remove any unbound streptavidin - alkaline phosphatase. To develop the plates, 100μl BCIP/NBT substrate solution (Thermo Scientific) was added to all wells. Plates were incubated in the dark for 10 minutes. Plate development was stopped by rinsing the plate 3 times with distilled water. The plate was left to dry overnight by placing it in the dark at room temperature. Spots representing IFN-γ-secreting cells were quantified by an automated CTL ELISpot reader (Immunospot) followed by manual quality control. An image of ELISpot results of a representative subject is shown in **Figure 1A**. Valid results were defined by a count of less than 100 SFC/10^6^ PBMCs in the negative control and more than 1000 SFC/10^6^ PBMCs in the positive control. For statistical analysis, a positive response was defined by 25 or more SFC/10^6^ PBMCs and by being greater than three-fold the background. T-cell reactivity was defined by the quantity of SFC/10^6^ PBMCs after subtracting the quantity SFC/10^6^ in the negative control wells (background signal).

### Statistical Analysis

Stata version 14 (StataCorp, USA) and GraphPad Prism software version 9 (GraphPad Software Inc, USA) were used for statistical analysis. Two tailed Mann-Whitney U testing was performed to assess the difference in magnitude of T-cell reactivity in terms defined as of Spot Forming Cells per one million PBMCs (SFC/10^6^ PBMC) between different permutations of HIV and cervical pathology status. Fisher’s exact test was used to test associations between each E6 or E7 HPV type-specific response and HIV infection. Spearman’s rank correlation test was used to determine the relationship between absolute CD4 counts and HIV viral load with HPV type specific T-cell reactivity. The definitions for a positive responses and T-cell reactivity have been provided in section above detailing IFN-γ release ELISpot assay

## Results

### Description of the Cohort

A complete summary of cervical diagnosis, HPV genotyping results and HIV-associated clinical parameters of study volunteers stratified by HIV status is provided in **Table 1**. In total, data from 373 women with median age of 41 (34 – 51 years interquartile range), cytohistological diagnosis of cervical lesions, known HIV status and valid ELISpot results were included in the statistical analyses presented herein. Cervical pathology data was available for all women included; 34% (126/373) had SCC, 12% (44/373) had HSIL/CIN2+, 11% (39/373) had LSIL/CIN1, 0.5% (2/373) had atypical cells of undermined significance and 43% (162/373) did not have any cervical lesions. HPV genotyping data was available for 343 women included in the present analysis: 107 where HPV16+, 49 HPV18+ and 38 women were HPV45 positive. Seventy six women were infected with one or more of the remaining 11 HR HPV types. One hundred women were infected with non-HR HPV types and 89 did not have any HPV infection.

Of the 202 women living with HIV, 75% were on ART, 34% had a CD4 count of 250 cells/µl or less and 34% of women had a HIV viral load of above 1000 RNA copies/ml (**Table 1**). In summary, the majority of women enrolled in this study were on ART and had a well-controlled HIV infection.

### Low Inherent Systemic Immunogenicity of HR HPV Oncoproteins

We first determined the magnitude of HPV-specific T-cell reactivity and proportions of HPV-oncoprotein-specific T cell responders in HIV negative women alone prior to determining the effect of HIV on the aforementioned parameters.

Overall, amongst all HIV negative women, the E6- and E7-specific T-cell reactivity was of low magnitude. The highest median SFC/10^6^ PBMCs for oncoprotein-specific T cells was 15 SFC/10^6^ PBMCs against HPV45-E6 (**Figure 1B**), this was significantly lower than the median magnitude of *M.tb*-specific T-cell reactivity (245 SFC/10^6^ PBMCs, p<0.001) and CMV-pp65-specific T-cell reactivity (median: 1412 SFC/10^6^ PBMCs, p<0.001). Amongst E6-specific T-cell reactivities, HPV16-E6 was the least immunogenic with median of 2.5 SFC/10^6^ PBMCs, this was half the median reactivity of HPV18-E6 (p = 0.008) and one sixth that of HPV45-E6 reactivity [p<0.001, (**Figure 1B**)]. An inverse pattern was observed for E7-specific T-cell reactivity. Here, HPV16-E7 had a low median reactivity of 5 SFC/10^6^ PBMCs, which was nonetheless double that of HPV18-E7, p = 0.012 and 5 times higher than HPV45-E7, p <0.001 (**Figure 1C**), but still below threshold for a positive response as defined by our study.

We then analyzed the proportions of HIV negative women with a positive E6-/E7-oncoprotein T-cell response. Even though HPV16 was the most frequently detected HPV type within the study population (**Table 1**), T-cell responses to HPV16-E6 were significantly less frequent (7%, 12 of 171 women), compared to T-cell responses to HPV18-E6 (15%, 25 of 171 women, p=0.04) and HPV45-E6 (41%, 65 of 160 women, p <0.001) (**Figure 1D**). In contrast, HPV16-E7-specific T-cell responses were detected in 23% (39 of 171 women), and hence more frequently detected as compared to HPV18 (11%, 20 of 171 women, p=0.07) and HPV45 (4%, 7 of 160 women, p<0.001). T-cell responses to the control antigens CMV-pp65 and *M.tb*-PPD were detected in most women at 99% (170/171, p<0.001) and 76% (130/171, p<0.001) respectively (**Figure 1E**).

These data demonstrate a low T-cell reactivity for HPV oncoproteins in peripheral blood for the clinically most relevant HR HPV types in adult HIV negative women and show that significant differences in immunogenicity exist between the oncoproteins of the three most relevant HPV types.

### HPV18 and 45 but Not HPV16 Infections Are Linked to Detectable E6- and E7-Specific T-Cell Responses Targeting the Infecting HPV Type

To determine the effect of prevalent HPV infection on the frequency of HPV-specific oncoprotein T-cell responses, we assessed whether HPV16, 18 or 45 infections in HIV- women were linked to detectable E6-/E7-specific T-cell responses against the autologous infecting HPV genotype in comparison to women not infected with either HPV16, 18 or 45, respectively.

**Figure 2:**
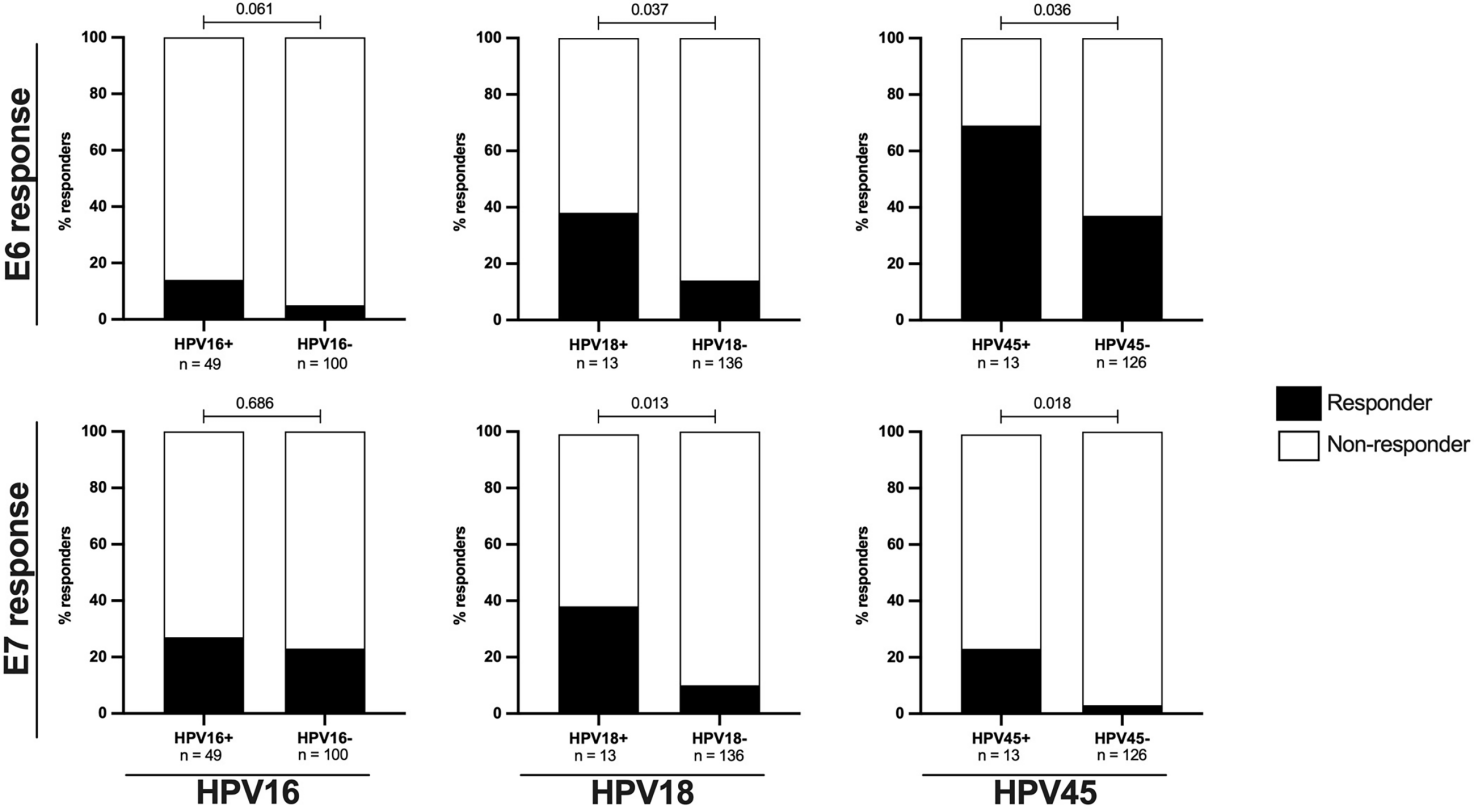
The proportions of E6/E7 HPV type specific oncoprotein T-cell responses in are increased in HIV- women with ongoing HPV infection. The proportion of HIV- women with HPV type specific oncoprotein T-cell responses in relation to HPV16, 18 or 45 infection status is shown as percentage. The upper panels presents data for E6 HPV type specific responses while the lower panel presents data for E7 HPV type specific responses. The black bars represent the percentage of responders while the white bars represent the percentage of non-responders, totaling to 100%. The n is given in the figure legend. Statistical analysis was performed using Fisher’s exact test, respective p-values are shown in the graph.

In HPV16 infected women only a non-significant increase in the frequency of HPV16-E6-specific responses was observed (**Figure 2**). 14% (7/49) of HPV16 infected women had HPV16-E6-specific T-cell responses compared to 5% of HPV16 negative women (5/100, p= 0.061). Women infected with HPV18 and 45 were significantly more likely to mount HPV type-E6-specific T-cell responses compared to those without these HPV infection (**Figure 2**); 38% (5/13) of HPV18 infected women responded to HPV18-E6 compared to 14% of HPV18 negative women (19/136, p = 0.037) and 69% of HPV45 infected women (9/13) responded to HPV45-E6 compared to 37% of HPV45 negative women (47/126, p = 0.036). A similar pattern was observed for E7-specific T-cell responses targeting autologous HPV type (**Figure 2**). Here the proportion of responders was 27% (13/49) for HPV16 infected women versus 23% (23/100) for HPV16 negative women (p = 0.686); 38% (5/13) for HPV18 infected women versus 10% (14/136) for HPV18 negative women (p = 0.013) and 23% (3/13) for HPV45 infected versus 3% (4/126) in HPV45 negative women (p = 0.018). When combining autologous-type-E6/E7-specific T-cell responses, the overall lowest frequency was detected in HPV16 infected women (33%, 16/49), as compared to women infected with HPV18 (46% (6/13), p = 0.516); and compared to women infected with HPV45 (69% (9/13), p = 0.026, data not shown). Taken together, these results show that prevalent HPV18 and 45, but not HPV16 infections, were linked to a significantly higher likelihood of mounting oncoprotein-specific T-cell responses against the infecting HPV type and suggest a particularly low inherent immunogenicity of HPV16 oncoproteins in direct comparison to the clinically relevant HR HPV types 18 and 45.

### Depletion of Oncoprotein-Specific T-Cell Responses Targeting the Infecting HR HPV Type in Women Living With HIV

To assess the effect of HIV infection on systemic HPV T-cell responses, we stratified the data into HIV– and HIV+ groups and subsequently analyzed the frequency of response and the magnitude of HR HPV oncoprotein-specific T-cell reactivity within these two groups.

Taking into account women with and without HPV infection, the frequency of E6-specifc T cell responses was comparable between HIV- and HIV+ women for HPV16 and HPV18 specific responses, but significantly differed for HPV45 specific responses (p = 0.006, **Table 2**). HIV infection further had no measurable effect on the proportion of HR HPV-E7-specific T-cell responses Also, the magnitude of HPV16- and HPV18-E6-specific T-cell reactivity was similar between HIV- and HIV+ volunteers, irrespective of whether or not the women had an ongoing HPV infection. Conversely, HPV45-E6-specific T-cell reactivity was significantly decreased in HIV+ women, p = 0.012 (**Supplementary Figure 1**). For E7-specific T-cell reactivity, HIV infection had no measurable effect on the magnitude of T-cell reactivity.(**Supplementary Figure 1B**).

**Table 2:** Proportions of E6- and E7-specific T-cell responses stratified by HIV status.

	E6-specific T cell response			E7-specific T cell response		
	% (n) HIV-	% (n) HIV+	p value	% (n) HIV-	% (n) HIV+	p value
HPV16 responders	7 (12/171)	8 (17/202)	0.700	23 (39/171)	24 (49/202)	0.807
HPV16 non-responders	93 (159/171)	92 (185/202)		77 (132/171)	76 (153/202)	
HPV18 responders	15 (25/171)	10 (21/202)	0.269	12 (20/171)	15 (31/202)	0.365
HPV18 non-responders	85 (146/171)	90 (181/202)		88 (151/171)	85 (171/202)	
HPV45 responders	41 (65/160)	26 (48/184)	0.006	4 (7/160)	8 (15/184)	0.187
HPV45 non-responders	59 (95/160)	74 (136/184)		96 (153/160)	92 (169/184)	

**Figure 3:**
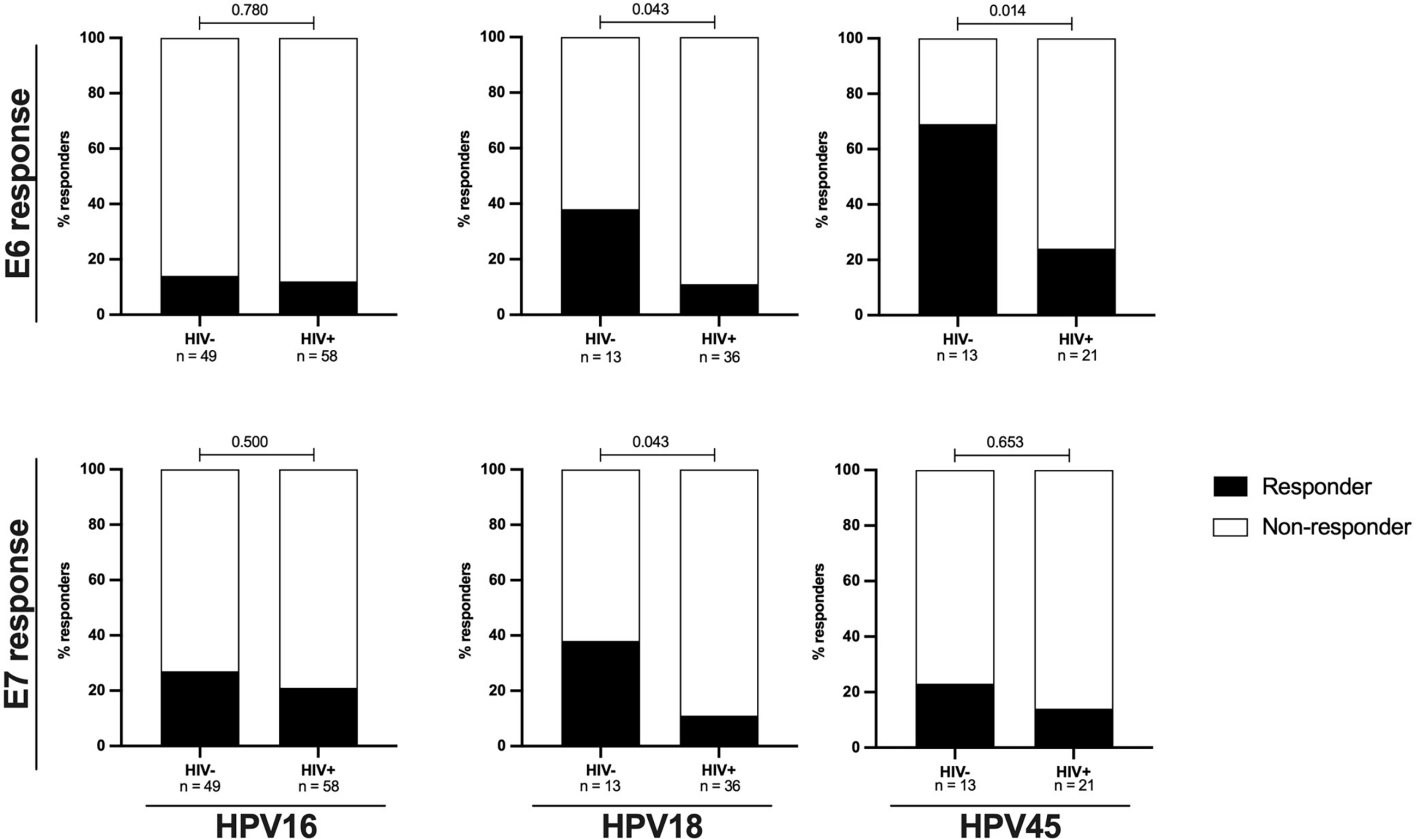
The proportions of autologous E6/E7 HPV type specific oncoprotein T-cell responses are reduced in HIV+ women. The proportion of women with autologous HPV16, 18 or 45 type specific T-cell responses stratified by HIV status is shown as percentage. Statistical analysis was performed using Fisher’s exact test, respective p-values are shown in the graph. The black bars represent the percentage of responders while the white bars represent the percentage of non-responders, totaling to 100%. The n is given in the figure legend. The upper panels presents data for E6 HPV type specific responses while the lower panel presents data for E7 HPV type specific responses.

**Figure 4:**
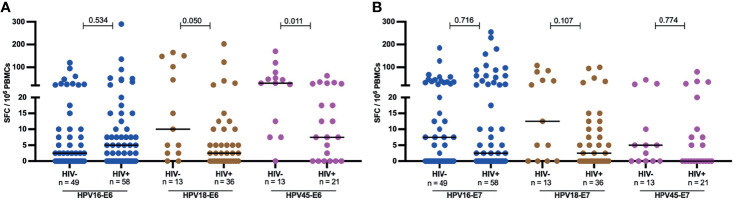
Magnitude of autologous HPV oncoprotein type specific T-cell reactivity. The magnitude of autologous T-cell reactivity in SFC/10^6^ against E6 **(A)** and E7 **(B)** HPV16, 18, 45 type specific oncoproteins stratified by HIV status. Each dot represents an individual study participant, median SFC/10^6^ PBMCs is indicated with black line in the graph and the n is indicated in the figure legends. Statistical analysis was performed using the Mann-Whitney U-test, the respective p-value is shown in the figures.

However, the effect HIV on the frequency of response and magnitude of HPV-specific T-cell reactivity was more apparent when the analysis focused on women with an ongoing HPV infection. Amongst HPV18 and HPV45 infected women, HIV infection was associated with diminished E6-specific T-cell responses targeting the infecting genotype. For HPV18-E6, only 11% (4/32) of HIV+HPV18+ women responded compared to 38% (5/13, p = 0.043) in HIV-HPV18+ women. Similarly, only 24% of HIV+HPV45+ women (5/21) responded to HPV45-E6 compared to 69% of HIV-HPV45+ women (9/13, p = 0.014) (**Figure 3**). In HPV16 infected women, autologous HPV16-E6-specific T-cell responses were rarely detected regardless of HIV co-infection, with 14% (7/49, HIV-) vs 12% (7/58, HIV+, p = 0.780) recognition. For autologous E7-specific T-cell responses, the response rates were low in HPV16 and 45 infected women regardless of HIV co-infection. However, HIV infection was again associated with significantly lower HPV18-E7-specific responses with 11% (4/36) vs 38% (5/13, p = 0.043) in HIV+ vs HIV- women, respectively (**Figure 3**). Analyses of autologous E6-/E7- T cell reactivity showed that E6-specific autologous reactivity was comparable between HIV- and HIV+ for HPV16. Conversely, median magnitude of T-cell reactivity for HPV18-E6 was 5-fold higher in HIV- when compared to HIV+ (p = 0.050), while reactivity for HPV45-E6 was 4-fold higher in HIV- as compared to HIV+ (p = 0.011, **Figure 4A**). There was no difference in the median magnitude of autologous E7-specifc T-cell reactivity for the three HR HPV types included in this analyses (**Figure 4B** **).**

### Depletion of HR HPV E6- and E7- Oncoprotein Specific T-Cell Responses in Women Living With HIV Is Linked to Low CD4 Counts and Detectable HIV Plasma Viremia

**Figure 5:**
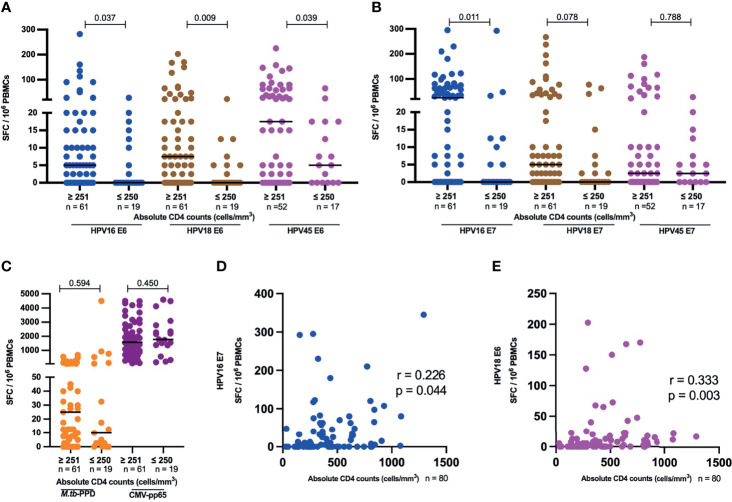
HPV-specific oncoprotein T-cell reactivity is reduced in HIV+ women with advanced HIV disease. The magnitude of reactivity against E6 **(A)** and E7 **(B)** HPV16, 18, 45 type specific oncoproteins **(C)** *M.tb*-PPD and CMV-pp65 is shown as SFC/10^6^ PBMCs and stratified by CD4 counts. Each dot represents an individual study participant, median SFC/10^6^ PBMCs is indicated with black line in the graph and the n is indicated in the figure legends. Statistical analysis was performed using the Mann-Whitney U-test. Correlation between absolute CD4 counts and HPV16 E7 **(D)** as well for HPV18 E6 **(E)** T-cell reactivity in SFC/10^6^ PBMCs is shown, correlation coefficients (r) and p-values are shown in the graphs. Statistical analysis was performed using the spearman’s ranks correlation test.

Since advanced HIV disease has been correlated with loss of pathogen-specific T cells, we analyzed HPV oncoprotein-specific T-cell reactivity in the context of HIV plasma viremia and CD4 T-cell depletion in HIV+ women with no cervical lesion stratified by HIV VL of less or greater than 1000 copies per ml and those with CD4 T-cells counts of less or greater than 250 cells/µl. These cut offs are aligned with WHO guidelines, which define immunological failure as CD4 T cell counts of 250 cells/mm^3^ or less and virological failure is defined as two sequential viral loads (VL) levels of 1000 or more copies/mL within 3 months (36). HIV+ women with > 250 CD4 T cells had significantly higher HPV E6-specific T-cell reactivity compared to those with ≤ 250 CD4 T cell counts/µl for HPV16, 18 and 45 (**Figure 5A**), with a 5-fold lower median for HPV16 (p = 0.037), 7-fold lower median for HPV18 (p = 0.009) and 3-fold lower median for HPV45 (p = 0.039). Similarly, E7-specific T-cell reactivity (**Figure 5B**), was higher in volunteers with > 250 CD4 T cells compared those with ≤ 250 CD4 T cells; 25-fold lower median for HPV16, p = 0.011 and 5-fold lower median for HPV18 reactivity, while no difference was observed for HPV45. Interestingly, no such differences were observed for *M.tb*-specific or CMV-specific T-cell reactivity (**Figure 5C**). In addition, we observed a positive correlation between absolute CD4 counts and HPV16 E7 as well as for HPV18 E6-specific T cell reactivity, r = 0.226 p = 0.044 and r = 0.333, p = 0.003, respectively (**Figure 5D, E**).

With respect to HIV viral load, HPV45 E6 T-cell reactivity was 2.3 times lower in samples which had ≥ 1001 HIV VL copies/mL compared to samples with ≤ 1000 (p = 0.029, **Supplementary Figure 2A**). There was no significant difference in E6-specific T-cell reactivity for HPV16 and 18 (**Supplementary Figure 2A**), and for E7-specific T-cell reactivity for HPV16, 18 and 45 (**Supplementary Figure 2B**). Interestingly, there was a weak negative correlation between HPV45 E6 T-cell reactivity and HIV RNA copies/ml, r = -0.273, p = 0.024 (**Supplementary Figure 2C**).

### Selective Depletion of HR HPV Oncogene-Specific T-Cell Responses in HIV+ Women With Precancerous Lesions or Cervical Cancer

**Figure 6:**
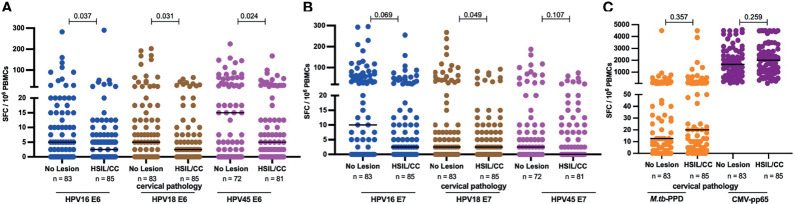
HPV-specific oncoprotein T-cell reactivity is reduced in HIV+ women with high grade intraepithelial lesions or cancer. The magnitude of reactivity against E6 **(A)** and E7 **(B)** HPV16, 18, 45 type specific oncoproteins **(C)** Mtb-PPD and CMV-pp65 is shown as SFC/10^6^ PBMCs and stratified by cervical pathology for these HIV+ women. Each dot represents an individual study participant, median SFC/10^6^ PBMCs is indicated with black line in the graph, p values are indicated in the graphs and the n is indicated in the figure legends. Statistical analysis was performed using the Mann-Whitney U-test.

To evaluate the possible link between precancerous and cancerous lesions with depleted HPV oncoprotein-specific T-cell reactivity in HIV+ women, we compared the magnitude of E6- and E7-specific T-cell reactivity in women with and without HSIL or SCC. HIV+ women with HSIL or SCC compared to those with no lesion had significantly lower median magnitudes of E6-specific T cells (**Figure 6A**); 2-fold less for HPV16- and 18-E6-specific T-cell responses (p = 0.037 and 0.031, respectively) and 3-fold less for HPV45-specific T cell responses, p = 0.024. No significant differences for E7-specific T-cell reactivity was observed for any of the three HPV types (**Figure 6B**) nor for control antigens *M.tb*-PPD and CMV-pp65 (**Figure 6C**).

## Discussion

In this study, we address the hypothesis that HIV-induced dysfunction or depletion of HR HPV-oncoprotein-specific T cells contributes to the increased risk for cervical cancer in HIV+ women. We analyzed T-cell responses targeting HPV types 16, 18 and 45, as these cause the vast majority of cervical cancer cases regardless of HIV infection or global region (11). We found that: a) HR HPV oncoproteins have a low inherent systemic immunogenicity with the lowest oncoprotein-specific T-cell reactivity observed for HPV16 - the most cancerogenic type; b) among HIV negative women, autologous HR HPV type-specific T-cell responses were significantly more frequently detected in women with a given HR HPV infection, compared to those without such an infection; c) HIV infection, advanced HIV disease progression and ongoing HIV viral replication were all linked to depletion of HR HPV-specific T-cell responses; d) Selective depletion of HR HPV-oncoprotein-specific T-cell reactivity was observed in HIV+ women with precancerous cervical lesions and cancer, whereas other pathogen-specific responses were preserved in these women. Together these results support the concept that depletion or dysfunction of HR HPV-specific T-cell responses predisposes HIV+ women to increased HPV persistence followed by the development of precancerous cervical lesions and cancer.

The very low inherent immunogenicity of HR HPV oncoproteins in our study confirms results from previous studies (38, 39). The non-cytopathic aviremic nature of the virus (40, 41) as well as the highly confined expression of the small E6 and E7 oncoproteins to the basal and parabasal cells of the cervical epithelium may all contribute to poor systemic immunogenicity of E6 and E7. It is noteworthy that HPV16 immunogenicity in the total cohort was particularly low when compared to HPV18 and HPV45. Indeed, in contrast to HPV18 and HPV45 infections, HPV16 infection was not linked to increased T-cell recognition of HPV16 E6 or E7. We therefore hypothesize that this particularly low inherent HPV16 oncoprotein immunogenicity plays a role for the remarkable persistence and cancerogenic potential of HPV16 infections as compared to other HR HPV types.

Systemic T-cell reactivity to the control antigens *M.tb*-PPD and CMV-pp65 was robust and as described before, HIV infection was associated with depletion of *M.tb*-specific, but not CMV-specific T-cell responses (26, 27). The latter were slightly increased in HIV infected women, confirming previous studies by our (26, 27) and other groups (42). The magnitude of E6 and E7 HR HPV type-specific systemic T-cell reactivity differed amongst HPV16, 18 and 45. For E6 peptides, the HPV16-E6 peptide was the least immunogenic while HPV45-E6 was the most immunogenic. Plausibly, the low immunogenicity of HPV16-E6 when compared to the HPV18-E6 and HPV45-E6 oncoproteins contributes to the cancerogenic potential of HPV16 cancers regardless of HIV infection. Interestingly, the converse was observed for E7 peptides, there, HPV16-E7 was the most immunogenic whereby HPV45-E7 was least immunogenic. It is worth reiterating that the median magnitude of these T-cell responses were below what is deemed a positive T-cell response. It is plausible that anti-E6 and E7- HPV systemic T-cell responses contribute differently to HPV clearance and cervical lesion regression. Tong et al., showed that E6, but not E7, systemic CD4 T-cell responses were associated with regression of anal high grade squamous intraepithelial lesions (43). Contrary, Seresini et al., have shown that PBMCs when stimulated with HPV18-E7 produce different CD4 cytokine profiles depending on whether the PBMC donor was HPV18+ or HPV18-. The aforementioned difference was not observed when the cells were stimulated with HPV18-E6 peptide (44). These findings are in line with our observation that E6 and E7 oncoproteins from the same HPV type differ in immunogenicity and may therefore have differing roles in the anti-HPV immune response. Nonetheless, grouping either E6 or E7 HPV type specific response as a response to a particular HPV type still indicated HPV16 as the least immunogenic and HPV45 as the most immunogenic.

HIV infection was associated with reduced frequency and magnitude of autologous HPV18- and 45-specific T-cell reactivity in HIV+ women, even though most of these women were on efficient ART. This implies that ART initiation may often not fully restore HPV-specific T-cell function which likely contributes to decreased HPV clearance and increased rates of cervical lesions. Indeed, most women living with HIV and diagnosed with cervical cancer in this study also were on ART treatment, which is consistent with other studies (45). Nicol and colleagues showed that while HPV infection increased T-cell infiltration and the secretion of IL-6, TNF-α and IFN-γ in cervical biopsies of HPV+ individuals, HIV-HPV co-infected individuals had significantly reduced amounts of IFN-γ, IL-6 and TNF-α (16), suggesting that HPV-specific T-cell responses are depleted or dysfunctional also within the mucosa.

Even though we have not observed any difference between HIV+ and HIV- women in frequency or reactivity of autologous T-cell immunity to HPV16, we find that women with advanced HIV disease (less than 250 CD4 T cells) and with no cervical lesions, had significantly lower immune reactivity against HPV16 than those with higher CD4 T-cell counts. This implies that advanced immunodeficiency rather than HIV positivity alone affects HPV16-specific T-cell reactivity, regardless of ART status. Indeed, ART status alone does not represent an adequate measure of immune reconstitution. Better measures would be: HIV viral suppression and reconstitution of peripheral and mucosal CD4 T-cells counts (36). Possibly, the majority of women who started ART before the *test and treat era* might not fully and adequately restore their HPV-specific mucosal immunity because of starting HIV treatment at a low nadir CD4 T-cell count (46, 47). Our results show that women with more advanced HIV disease had significantly lower magnitudes of HPV-oncoprotein-specific T-cell reactivity compared to those with less advanced HIV progression. The combined data therefore links the increased risk for HR HPV infection and associated cervical disease caused by HIV infection – particularly evident in women with more advanced acquired immunodeficiency - to dysfunction and depletion of HPV-specific T cell immunity. Indeed, a large number of studies have reported an inverse relationship between absolute CD4 T-cell counts and HPV infection rates and subsequent development of pre-cancerous lesions and SCC (48–50). In this context, it will be important to clarify whether early ART treatment initiation, when CD4 T-cell counts are still high, may counteract HIV-induced T-cell dysfunction, reduce risk for HPV virus persistence thereby reducing the risk of cervical cancer. The importance of CD4 T cells with respect to HPV infection and disease has been strengthened by Steele et al., showing in their study that even though the frequency of systemic anti-HPV CD8 T-cell responses was twice that of CD4 T-cell responses, CD4 T-cell reactivity was reduced in patients with advanced lesions, implying that CD4 T cells are crucial in HPV clearance and cervical lesion regression (38). Similarly, Coleman et al., have shown that regressing genital warts are predominantly infiltrated by activated memory CD4 T cells, further underpinning the importance of CD4 T cells (12).

Furthermore, we report that women living with HIV and with high-grade intraepithelial cervical lesions or cancer (HSIL/SCC) had significantly lower systemic HR HPV oncoprotein T-cell reactivity compared to those without cervical lesions. Cervical lesions (especially cancer) are associated with T-cell exhaustion, T-cell activation and inflammation (51–53). Capacity for HPV-specific IFN-γ production by T cells may therefore continuously be reduced as cervical lesion progress (and more oncoproteins are being expressed). Together these data are consistent with the hypothesis that HIV infection and lesions act synergistically to enhance immune dysfunction of the host response to HPV infection, which in turn counteracts HPV clearance and accelerates cancerogenesis.

Quantifying systemic T-cell responses to HR HPV oncoproteins is challenging as the lack of a viremic phase and the numerous immune evasion mechanisms of HPV negatively impact systemic HPV-specific T-cell reactivity. To overcome this challenge, different studies have modified their ELISpot assays in a variety of ways: a) increasing the time to culture during the *ex vivo* re-stimulation (3 – 9 days) (21, 54, 55), b) co-culturing lymphocytes with IL-2 for 9 days (55), or c) pulsing autologous dendritic cells prior to long term stimulation (56). In different studies the final peptide concertation ranges from 2µg/ml to 10µg/ml. While these approaches may have their merits, our approach of direct short-term ex vivo re-stimulation of freshly isolated PBMC with HR HPV type specific peptides has the advantage of closely reflecting systemic T-cell reactivity *in vivo*. Due to the infrequent nature of systemic HPV responses, especially in HIV+ women, we could not analyze the effects of CD4 levels, HIV RNA plasma levels as well as cervical pathology on autologous systemic HPV-specific T-cell immunity. However, we believe that effects of the aforementioned parameters on HPV-specific immunity is consistent with the results presented regardless of presence of an ongoing HPV infection.

In conclusion, we observed that HIV-associated depletion of HR HPV oncoprotein-specific T-cell reactivity is particularly pronounced in patients with progressed, viremic HIV infection and in those with HPV associated premalignant and malignant cervical lesions. This low HR HPV-oncoprotein-specific T-cell reactivity likely contributes to the increased HR HPV persistence, cervical lesion progression and accelerated cancer development in HIV+ women.

## Data Availability Statement

The raw data supporting the conclusions of this article will be made available by the authors, without undue reservation.

## Ethics Statement

The studies involving human participants were reviewed and approved by the Mbeya Medical Research and Ethics review Committee (MRH/R.10/8/Vol. VI/107), the Tanzanian National Health Research Ethics Committee (NIMR/HQ/R.8a/Vol. IX/1422) and the Ethics Committee of the Medical Faculty of University of Munich (project ID: 308-11). The patients/participants provided their written informed consent to participate in this study.

## Author Contributions

Conceptualization, CG, AK, and RK. Formal analysis, WM, KH, MC, and CG. Clinical investigation, AK, RM, MS, FR, and LT. Laboratory investigation, WM, AH, JaM, JoM, AM, MM, WoM, RK, LT, MC, and CG. Resources, MH, LM, FR, LT, and RK. Data curation, PA and ES. Supervision, AK, KH, RM, MH, LM, OG, MC, and CG. Project administration, AK, RM, MH, LM, OG, and CG. Funding acquisition, CG, AK, MH, and LM. All authors contributed to writing and review of the manuscript.

## Funding

This study was funded by the DFG African cooperation projects in Infectiology (grant 2128/2-1).

## Conflict of Interest

The authors declare that the research was conducted in the absence of any commercial or financial relationships that could be construed as a potential conflict of interest.

## Publisher’s Note

All claims expressed in this article are solely those of the authors and do not necessarily represent those of their affiliated organizations, or those of the publisher, the editors and the reviewers. Any product that may be evaluated in this article, or claim that may be made by its manufacturer, is not guaranteed or endorsed by the publisher.

## Supplementary Material

Supplementary Figure 1 HIV status does not affect magnitude of HPV type specific oncoprotein T-cell reactivity. The magnitude of T cell reactivity against E6 **(A)** and E7 **(B)** HPV16, 18, 45 type specific oncoproteins is shown as SFC/10^6^ PBMCs and stratified by HIV status. Each dot represents an individual study participant, median SFC/10^6^ PBMCs is indicated with a black line in the graph, p values are indicated in the graphs and the n is indicated in the figure legends. Statistical analysis was performed using the Mann-Whitney U-test

Supplementary Figure 2 HPV45 E6-specific oncoprotein T-cell reactivity is reduced in HIV+ with elevated HIV RNA copies/ml. The magnitude of reactivity against E6 **(A)** and E7 **(B)** HPV16, 18, 45 type specific oncoproteins among HIV+ women is shown as SFC/10^6^ PBMCs and stratified by HIV viral load. Each dot represents an individual study participant, median SFC/10^6^ PBMCs is indicated with a black line in the graph, p values are indicated in the graphs and the n is indicated in the figure legends. Statistical analysis was performed using the Mann-Whitney U-test. Correlation between HIV copies/ml and HPV45 E6 T cell reactivity in SFC/10^6^ PBMCs **(C)** is shown, correlation coefficients and p values are shown in the graphs. Statistical analysis was performed using the spearman’s ranks correlation test.
