# Supplementary figures and images for: Depletion of Human Papilloma Virus E6- and E7-Oncoprotein-Specific T-Cell Responses in Women Living With HIV
Source: Front Immunol. 2021 Oct 25;12:742861. doi: 10.3389/fimmu.2021.742861 (PMC8573218; doi:10.3389/fimmu.2021.742861)

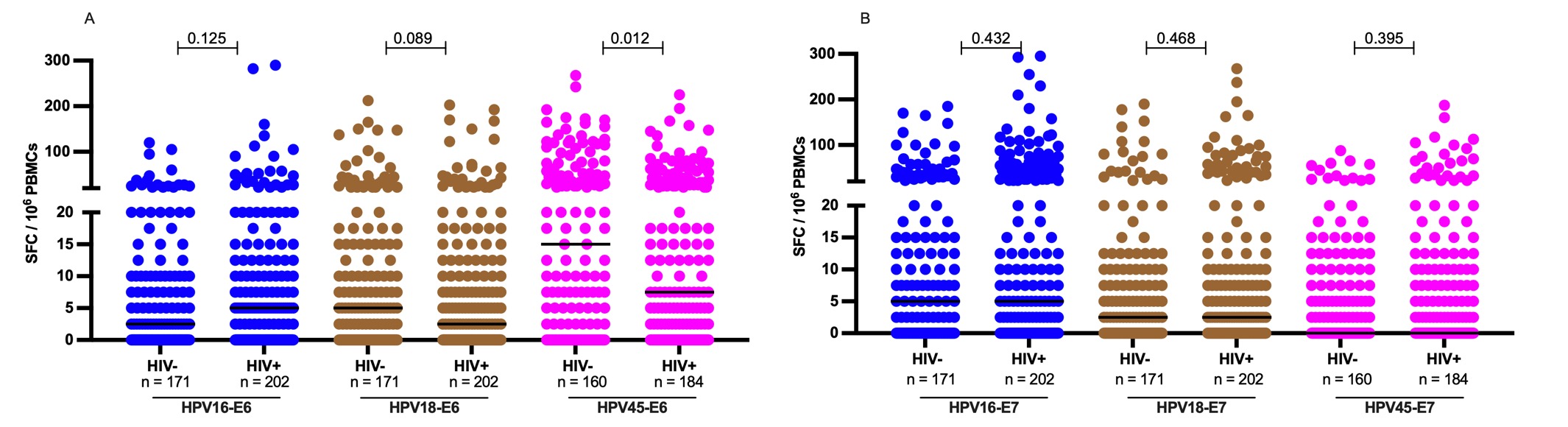

Supplement: Supplementary Figure 1 — HIV status does not affect magnitude of HPV type specific oncoprotein T-cell reactivity. The magnitude of T cell reactivity against E6 (A) and E7 (B) HPV16, 18, 45 type specific oncoproteins is shown as SFC/106 PBMCs and stratified by HIV status. Each dot represents an individual study participant, median SFC/106 PBMCs is indicated with a black line in the graph, p values are indicated in the graphs and the n is indicated in the figure legends. Statistical analysis was performed using the Mann-Whitney U-test [file Image_1.jpeg]

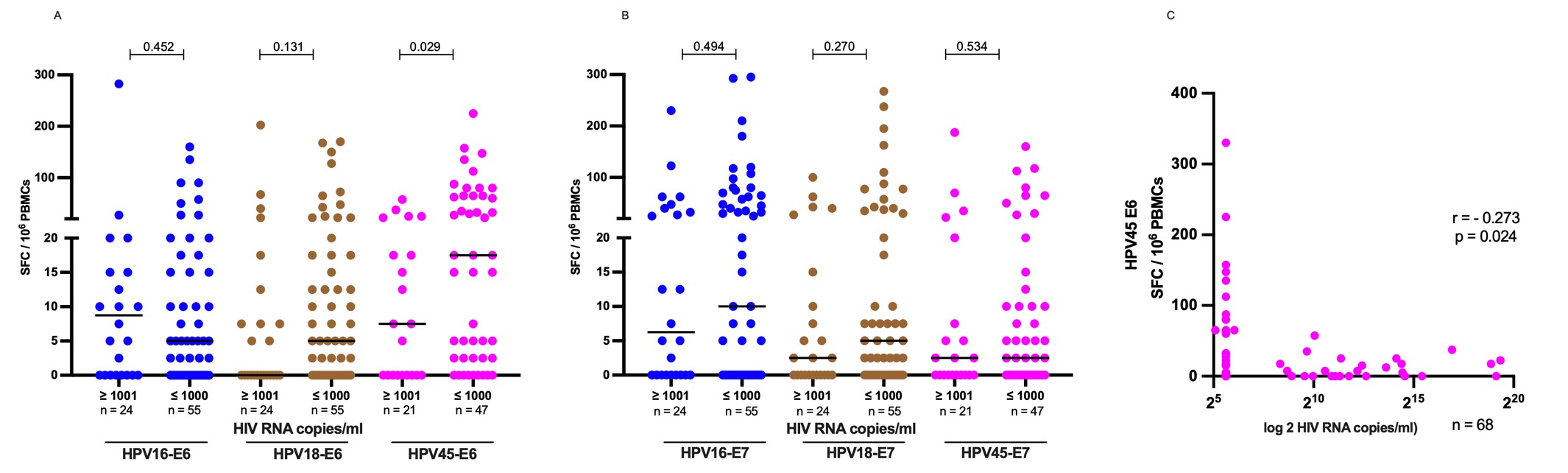

Supplement: Supplementary Figure 2 — HPV45 E6-specific oncoprotein T-cell reactivity is reduced in HIV+ with elevated HIV RNA copies/ml. The magnitude of reactivity against E6 (A) and E7 (B) HPV16, 18, 45 type specific oncoproteins among HIV+ women is shown as SFC/106 PBMCs and stratified by HIV viral load. Each dot represents an individual study participant, median SFC/106 PBMCs is indicated with a black line in the graph, p values are indicated in the graphs and the n is indicated in the figure legends. Statistical analysis was performed using the Mann-Whitney U-test. Correlation between HIV copies/ml and HPV45 E6 T cell reactivity in SFC/106 PBMCs (C) is shown, correlation coefficients and p values are shown in the graphs. Statistical analysis was performed using the spearman’s ranks correlation test. [file Image_2.jpeg]
